# Myasthenia Gravis following Low-Osmolality Iodinated Contrast Media

**DOI:** 10.1155/2014/963461

**Published:** 2014-12-03

**Authors:** Luca Bonanni, Michele Dalla Vestra, Andrea Zancanaro, Fabio Presotto

**Affiliations:** Department of Internal Medicine, “Dell'Angelo” General Hospital, 11 Paccagnella Street, Mestre, 30171 Venice, Italy

## Abstract

We describe the case of 79-year-old man admitted to our general hospital for a 6-week history of progressive dysphagia to solids and liquids associated with weight loss. To reach a diagnosis a total body CT scan with low-osmolality iodinate contrast agent was performed. Two hours later the patient developed an acute respiratory failure requiring orotracheal intubation and mechanical ventilation. The laboratory and neurological tests allow formulating the diagnosis of myasthenia gravis. In literature, other three case reports have associated myasthenic crisis with exposure to low-osmolality contrast media. This suggests being careful in administering low-osmolality contrast media in myasthenic patients.

## 1. Introduction

Myasthenia gravis is often underrecognised in the elderly people, partly because symptoms such as dysphagia, fatigue, and slurred speech can have a broad differential diagnosis  [1]. CT scan with iodinated contrast media is often performed to investigate symptoms connected with this disease (like weight loss).

## 2. Case Presentation

In April 2013, a 79-year-old man was admitted to our general hospital for a 6-week history of progressive dysphagia to solids and liquids associated with weight loss of 3 kg. He denied any other symptoms. His past medical history included type II diabetes, hypertension, and COPD. He could walk and neurological examination showed no ocular symptoms or muscle weakness. There was a mild dehydration, but examination of the cardiovascular and abdominal systems was unremarkable. Investigations showed a mild increase of serum creatinine (1.3 mg/dL). Gastroscopy showed a mild hyperemic duodenitis: for this reason a therapy with Pantoprazole was started.

During the stay in our ward he developed a progressive asthenia, leading to inability to walk and to lift the head to watch the examiner without using his hand. A blood sample was taken to test for acetylcholine-receptor antibodies. The same day, a total body CT scan with low-osmolality iodinate contrast agent (Iohexol, concentration 300 mgI/mL, volume 200 mL) was performed. Two hours later the patient developed an acute respiratory failure requiring orotracheal intubation and mechanical ventilation. In the Intensive Care Unit the weaning of mechanical ventilation was soon initiated and after two days the extubation was performed. After the extubation the patient showed complete dysphagia, dysarthria, hypophonia, and proximal muscle hyposthenia. While waiting for the serologic test, pyridostigmine was given: there was a marked improvement in the following 24 hours with a complete neurological recovery. The diagnosis of myasthenia gravis was confirmed by the acetylcholine-receptor antibody test, by the repetitive nerve stimulation, and by the single fibre electromyography.

Before the CT scan, the patient never experienced respiratory symptoms (dyspnea or orthopnea) and there were no signs of a reexacerbation of COPD (no productive cough; the chest examination was negative for rumors, the respiratory rate was always in the normal range, and the oxygen saturation ranged from 94 to 96 in RA). No new drug was added to the therapy with Pantoprazole (that had been administered daily for ten days before the acute respiratory event and it is still ongoing). Our diagnosis was a myasthenic crisis induced by the low-osmolality iodinate contrast agent.

## 3. Discussion

Many drugs can unmask or exacerbate myasthenia gravis, among them the iodinated contrast agents. There have been a few case reports and case series describing isolated examples of a myasthenic crisis after exposure to intravenous high-osmolality iodinate material, but, to our knowledge, only three other case reports have associated myasthenic crisis with exposure to low-osmolality contrast media  [2, 3].

This suggests being careful in administering low-osmolality contrast media in myasthenic patients.

We also think that myasthenic crisis induced by contrast media is underrecognised. In our opinion the diagnosis of myasthenic crisis should always be considered in the presence of respiratory symptoms after contrast media to define the real size of the problem and evaluate the opportunity to perform a noncontrast CT scan or a MRI. If no other options are possible, we suggest premedicating myasthenic patients before CT scan with iodinated contrast media.
